# Spontaneous Epidural Haematoma and Sigmoid Sinus Thrombosis due to Pyogenic Mastoiditis in Children: A Case Report

**DOI:** 10.1155/crdi/6482086

**Published:** 2025-12-22

**Authors:** Dionysia Fermeli, Eirini Kostopoulou, Panagiotis Tsipouriaris, Sotirios Raftopoulos, Andreas Theofanopoulos, Polyniki Sotiria Liapikou, Georgia Markou, Despoina Gkentzi, Gabriel Dimitriou

**Affiliations:** ^1^ Department of Neurosurgery, University Hospital of Patras, Patras, Greece, pgnp.gr; ^2^ Department of Pediatrics, University Hospital of Patras, Patras, Greece, pgnp.gr

**Keywords:** case report, epidural haematoma, hydrocephalus, mastoiditis, sigmoid sinus thrombosis

## Abstract

**Background:**

Mastoiditis represents the most common complication of acute otitis media, particularly during the first years of life. Epidural haematomas refer to a life‐threatening condition due to the extra‐axial collection of blood between the dura matter and the inner table of the skull, more frequently caused by head trauma. In the absence of head trauma, the epidural haematoma is called spontaneous. Sigmoid sinus thrombosis is a rare complication of mastoiditis. A case of a child with an atypical presentation of acute otitis media and mastoiditis and a rare combination of complications, such as spontaneous epidural haematoma secondary to sigmoid sinus thrombosis, is presented.

**Case Report:**

We present a 4‐year‐old boy with high fever, vomiting, temporal headache and altered mental status. On repeat efforts to evaluate the tympanic membranes due to poor cooperation, a bulging, inflamed and opacified tympanic membrane alongside an attenuated light reflex were identified in the left ear, confirming the diagnosis of acute otitis media. No redness or swelling of the mastoid process was observed. Due to the worsening mental status, a CT head scan was performed, which revealed left sigmoid sinus thrombosis, an epidural collection and hydrocephalus. The patient underwent urgent extraventricular drain insertion, a left lateral suboccipital craniotomy and evacuation of the epidural collection, which was proven to be a haematoma. After 1 month of intravenous antibiotics and hospitalization, he was discharged home asymptomatic and with no neurological deficits.

**Conclusion:**

We present a case of atypically presenting mastoiditis secondary to otitis media, further complicated by the development of sigmoid sinus thrombosis and epidural haematoma. We highlight the importance of regular physical evaluations in young patients with poor cooperation. Also, in the presence of mastoiditis and neurological symptoms, a brain image scan is crucial for the diagnosis of potentially fatal complications.

## 1. Introduction

Acute mastoiditis is the most common complication of acute otitis media [1]. If left untreated, it can result in dangerous, potentially fatal sequela, such as meningitis, venous sinus thrombosis and intracranial abscess [1]. With the advent of antibiotics, mastoidectomy and antipneumonococcal vaccination, the risk of acute otitis media progressing to mastoiditis as well as the mortality rate of mastoiditis have declined significantly [1].

We present the case of a child with acute otitis media complicated by acute mastoiditis and intracranial complications, such as spontaneous epidural haematoma (SEDH), sigmoid sinus thrombosis and obstructive hydrocephalus. Through this case, we aim to raise awareness concerning the likelihood of delayed identification of acute otitis media and mastoiditis due to atypical clinical presentation or rapid clinical escalation that may result in severe intracranial complications.

The research complies with the guidelines for human studies and was conducted ethically in accordance with the World Medical Association Declaration of Helsinki. Written informed parental consent was obtained from the patient’s parents for publication of this case report and any accompanying images. The study was approved by the Research Ethics Committee of the University Hospital of Patras (Approval number: 64904).

## 2. Case Presentation

### 2.1. History and Presentation

A 4‐year‐old boy was transferred to the Paediatric Emergency Department of our University Hospital from a district hospital in October 2023 due to high fever, vomiting, temporal headache and altered mental status. The patient had been febrile (up to 39.5°C) for two days before visiting the district hospital, showing an increasing intensity and frequency of the fever. Soon after the onset of fever, he complained of persisting temporal headache and fatigue, and few hours later he started vomiting and refused to drink or eat. Subsequently, nearly 24 h after the onset of fever, he became drowsy and continued to deteriorate over the last 8 h prior to admission. Previous medical history was unremarkable with no history of head trauma.

Upon admission, the patient was apyrexial and hemodynamically stable, mildly dehydrated and lethargic, but arousable. He had orientated speech, a Glasgow Coma Scale of E3V5M6, and showed no nuchal rigidity or Kernig and Brudzinski signs. His pupils were equal and reactive to light, the tendon reflexes were normal, and no focal neurological signs were identified. Cranial nerve examination was normal and no signs of raised intracranial pressure, such as ocular disturbances, were identified. Redness of the pharynx, absence of nasal congestion and symmetrical breathing sounds without respiratory distress were also present on examination. Abdominal examination was normal and there was no skin rash. A thorough ear examination was not possible initially due to poor patient cooperation and narrowing of the external auditory canal of the left ear which impeded the view of the tympanic membrane. However, upon repeated inspection, the tympanic membrane appeared bulging, erythematous and opacified and exhibited loss of the normal light reflex, confirming the diagnosis of acute otitis media. Although otalgia was not mentioned among the initially reported symptoms, upon repeat questions the mother remembered the child complaining of otalgia on one occasion soon after the onset of fever. Examination of the mastoid process showed no redness or swelling. Nonetheless, the presence of tenderness could not be assessed due to poor cooperation, thus acute mastoiditis could not be excluded.

His laboratory tests showed elevated white blood cell count (WBC: 24.10 Κ/μL) with raised neutrophil count (89%), elevated C‐reactive protein levels (CRP: 20.26 mg/dL, normal range < 0.6) and platelet count (PLT: 524 Κ/μL, normal range < 400). Haematocrit decreased from 34.1% at the territory hospital to 29.2% in our hospital within few hours. Coagulation tests were within normal range, and the rest of the blood tests were unremarkable.

**Figure 2:**
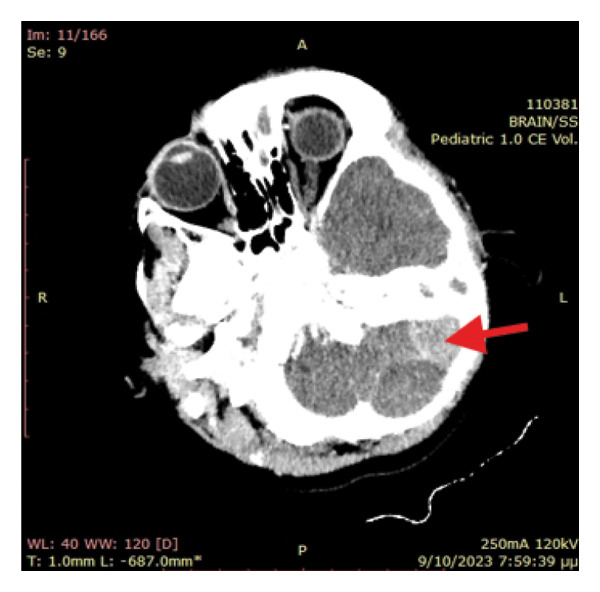
Hyperdense formation of the thrombosed left sigmoid sinus.

The primary suspected diagnoses included septicaemia and central nervous system (CNS) infection; therefore, treatment with ceftriaxone, clindamycin and acyclovir were initiated. Subsequently, an urgent head CT scan was ordered, as the patient was gradually drowning from GCS 14/15 to a comatose state accompanied with episodes of vomiting. Emergency pathologies such as an acute haemorrhage or cerebral oedema were excluded. However, a hyperdense (∼60HU) extraparenchymal formation located at the anatomical position of the sigmoid sinus and measuring 25 × 18 mm was identified, together with a hypodense extradural collection of 50 × 25 mm in contact with the left transverse venous sinus. Mass effect on the fourth ventricle, remarkably dilated third and lateral ventricles and dislocation of the medulla to the right were also observed, suggesting acute obstructive hydrocephalus (Figure 1). Opacification of the mastoid air cells and inner ear were also present, as of acute mastoiditis and otitis, without erosion of the mastoid bony septa. A CT scan with intravenous contrast agent (CT Venography) was subsequently performed showing lack of sketching of the left sigmoid sinus as of sinus thrombosis (Figure 2).

Figure 1(a) Obstructive hydrocephalus with enlargement of the 3^rd^ and lateral ventricles. (b) Extradural collection of the left posterior fossa (red arrow) and complete elimination of basal cisterns and 4th ventricle (yellow arrow) causing severe compression to the brainstem.

**Figure:**
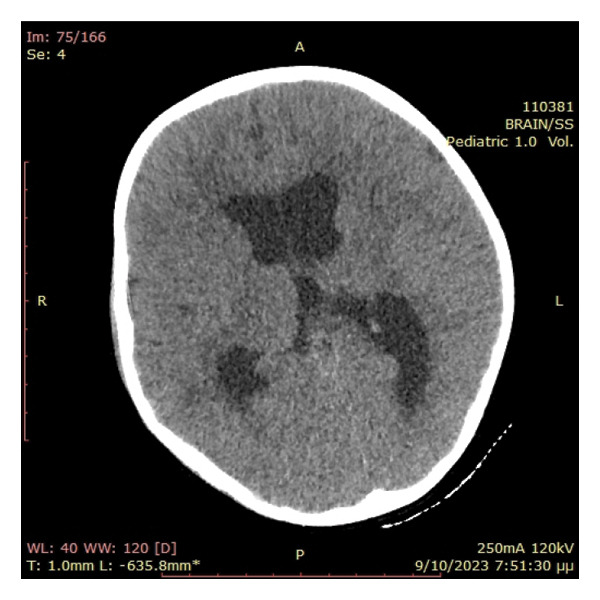
(a)

**Figure:**
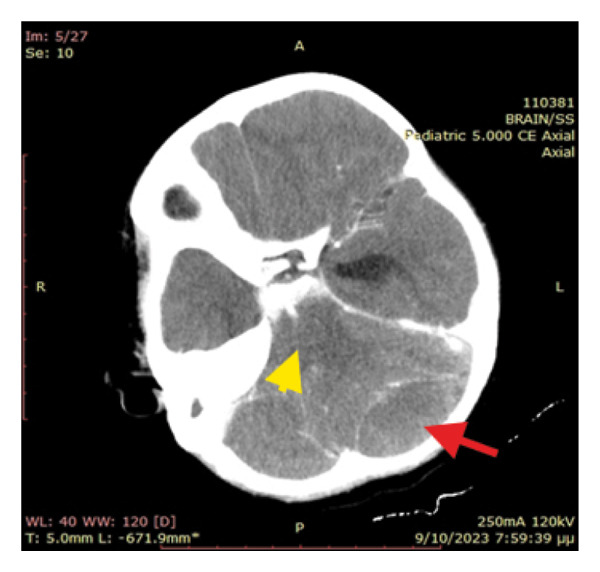
(b)

### 2.2. Operation and Postoperative Care

The patient was intubated at a GCS of 8/15 nearly 1.5 h post admission and transferred immediately to the neurosurgery operating theatre. An extraventricular drain (EVD) was inserted through the left Frazier point and afterwards, the patient underwent a left lateral suboccipital craniotomy and complete evacuation of the epidural collection, which turned out to be a haematoma. Lab tests of the cerebrospinal fluid (CSF) were unremarkable (clear colourless appearance, WBC: 9, RBC: 1950, glucose: 72 mg/dL, and protein: 7.1 mg/dL). Ziehl‐Nielsen and Gram stains were negative. Blood and CSF cultures were also obtained. The patient remained intubated and was transferred to the paediatric intensive care unit (ICU) for further treatment.

On the first postoperative day, an MRI head scan was conducted, which showed complete evacuation of the epidural haematoma, basal cisterns without oedema or compression, and no image of hydrocephalus as the EVD was placed directly into the triangle of the left lateral ventricle (Figure 3). Moreover, the MRI depicted better left sigmoid thrombosis in addition to ipsilateral pyogenic mastoiditis. CSF cultures showed multisensitive *Streptococcus sanguinis*. On the same day, the patient was transferred to another University Children’s Hospital for ENT evaluation and management. The diagnosis of acute mastoiditis was further substantiated by a temporal bone CT scan, after which a mastoidectomy was performed. Intravenous antibiotics were continued for 4 weeks and low‐molecular weight heparin was administered for 3 months. After 1 month of hospitalisation, the patient was discharged home asymptomatic and with no neurological deficits.

Figure 3(a) T1 sequence showing complete evacuation of the epidural haematoma (yellow arrow) and decompression of the brainstem and basal cisterns (red arrow). (b) T1 sequence showing the EVD catheter without hydrocephalus.

**Figure:**
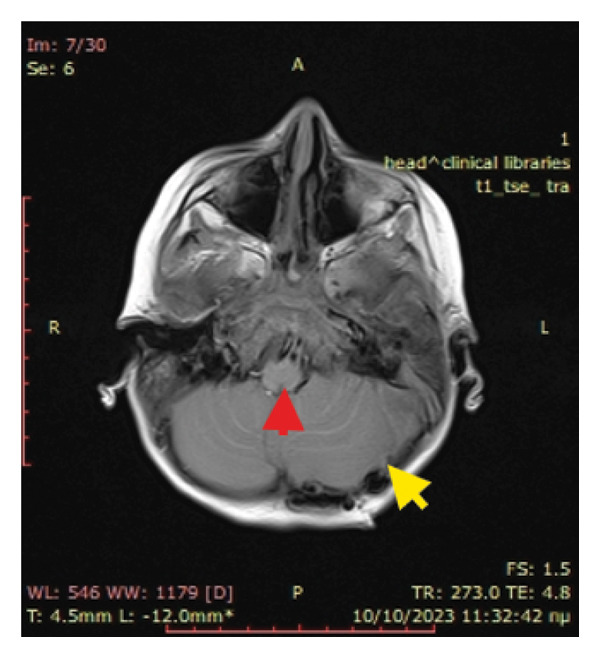
(a)

**Figure:**
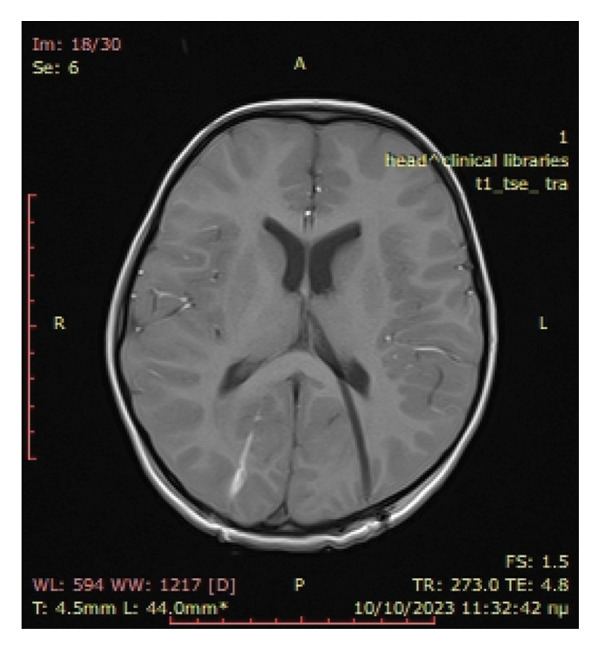
(b)

## 3. Discussion

Mastoiditis represents the most common complication of acute otitis media [2]. The pediatric population, particularly the first years of life, is more prone to mastoid infection due to anatomical, immunological and infectious conditions [1].

The main pathogen of acute mastoiditis is *Streptococcus pneumoniae*, followed by *Streptococcus pyogenes*, *Fusobacterium necrophorum*, *Haemophilus influenzae and Staphylococcus aureus*. In the postantibiotic and postvaccination era, the disease is rare but remains dangerous, often requiring prolonged antimicrobial and surgical approach to avoid severe complications, which can sometimes be devastating and lethal [1].

Clinical presentation includes lethargy (96%), abnormal tympanic membrane (82%), retroauricular skin erythema, tenderness and oedema and proptosis of the auricle (80%). Fever (76%), narrowing of the external auditory canal (71%), otalgia (67%) and otorrhoea (50%) are also common clinical features [1]. Of note, as opposed to common presentation, retroauricular inflammation and otalgia are not always present. In the present case, retroauricular inflammation was absent, which in association with the patient’s young age, thus poor cooperation and limited capacity to accurately describe his symptoms such as otalgia, posed a challenge for diagnosis.

Antibiotics represent the first‐line treatment for mastoiditis, whereas in further complicated cases, medications such as anticoagulants and/or corticosteroids may be added. In severe forms of the disease, surgical treatments, such as mastoidectomy, incision of abscesses and neurosurgical procedures, may also be required [1].

Otitis media and associated mastoiditis can cause sigmoid sinus thrombosis due to the extension of inflammation from the ear cavity to the mastoid bone, which is in close relationship to the sinus wall and the propagation of infection from the small venules draining the mastoid cavity into the sigmoid sinus [3, 4]. Increased clinical vigilance and availability of screening tests (CT and MRI scans) facilitate the diagnosis of sigmoid sinus thrombosis.

In our case, image studies revealed sigmoid sinus thrombosis and an adjacent posterior fossa epidural haematoma causing obstructive hydrocephalus due to mass effect. The CT scan showed an isodense extradural collection of the left posterior fossa (Figure 1). Initially, it was perceived as an epidural abscess because acute epidural haematomas are expected to be hyperdense. The craniotomy revealed only epidural blood and as a result, the isodensity suggests that it was a hyperacute epidural hematoma. There was neither intraoperative active bleeding nor a sign of empyema or abscess. A small amount of the epidural blood was sent to the lab without any microbial growth. CTV showed left sigmoid sinus lack of sketching, suggesting sinus thrombosis.

In addition, SEDHs are generally rare entities and in the absence of head trauma, infection is a potential cause [5]. They are also associated with coagulation disorders and dural vascular malformations [6, 7]. The incidence of SEDHs is unknown [3]. Anterior cranial fossa is the most frequent site of SEDHs due to craniofacial infections, whereas mastoiditis is the main cause for posterior fossa SEDHs [8].

Epidural haematomas can be caused by either the spread of the inflammatory factors to the vessels which makes them more vulnerable to bleed after some minor trauma or the dural detachment from the inner table as a result of infection products such as pus, air and exudates [7]. Furthermore, otitic hydrocephalus has been described as a rare complication of acute or chronic otitis media, often associated with lateral sinus thrombosis [9]. Potential mechanisms include reduced CSF resorption due to the thrombosis, which increases the CSF pressure, and impaired venous drainage [7].

## 4. Conclusion

We present a case of acute otitis media and multiple complications occurring simultaneously. The importance of a high index of suspicion for ear disease in the absence of an obvious history or examination findings, as well as of regular physical evaluations in young patients with poor cooperation is emphasized. In the presence of acute otitis media and neurological symptoms (headache, vomiting and confusion), a brain image scan is of the utmost importance so that conditions such as sigmoid sinus thrombosis and haemorrhages are ruled out.

### 4.1. Learning Points

• Otitis‐associated mastoiditis is a diagnosis that may be overlooked in the absence of a reliable medical history or clinical indications of mastoid inflammation.
• The presented case indicates that mastoiditis may not only have atypical presentation but can also be associated with rare complications such as sigmoid sinus thrombosis and epidural haematoma.
• Increased clinical vigilance and availability of screening tests (CT and MRI scans) facilitate the diagnosis of rare yet potentially lethal complications, such as venous sinus thrombosis and haemorrhage.

## Ethics Statement

This study was performed in line with the principles of the Declaration of Helsinki.

## Consent

Written informed consent was obtained from the patient’s parents for anonymised patient information to be published in this article.

## Conflicts of Interest

The authors declare no conflicts of interest.

## Author Contributions

Dionysia Fermeli and Eirini Kostopoulou share first authorship.

## Funding

No funding was received for this study.

## Data Availability

The data that support the findings of this study are available from the corresponding author upon reasonable request.
